# Supplementary material for: Evaluation of the genetic risk for COVID-19 outcomes in COPD and differences among worldwide populations
Source: PLoS One. 2022 Feb 23;17(2):e0264009. doi: 10.1371/journal.pone.0264009 (PMC8865687; doi:10.1371/journal.pone.0264009)

S1 Fig. Allelic frequencies for significant SNPs, for A - susceptibility (rs286914 and rs12329760) and B - severe response (rs657152 and rs11385942) to COVID-19 infection. No significant differences were found between Control group of Baixo Vouga cohort and Minho cohort for any of the tested SNPs; rs286914: p-value=0.54; rs12329760: p-value=0.06; rs657152: p-value=0.44. rs11385942 was not evaluated in Minho Cohort.

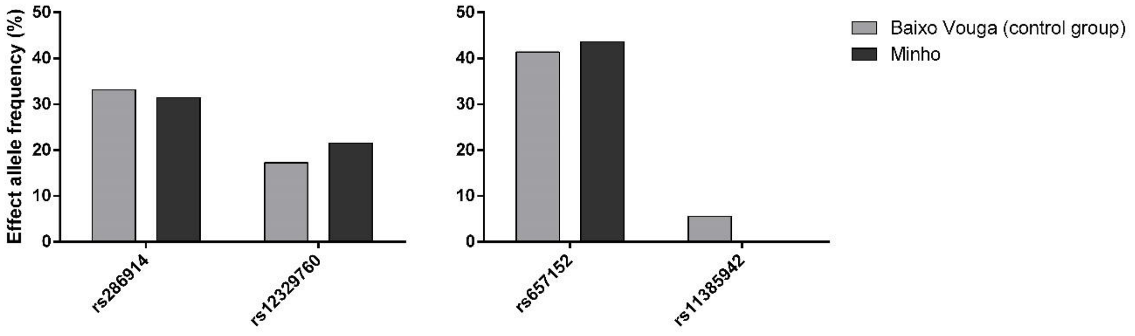

Supplement: S1 Fig — Allelic frequencies for significant SNPs, for A—susceptibility (rs286914 and rs12329760) and B—severe response (rs657152 and rs11385942) to COVID-19 infection. No significant differences were found between Control group of Baixo Vouga cohort and Minho cohort for any of the tested SNPs; rs286914: p-value = 0.54; rs12329760: p-value = 0.06; rs657152: p-value = 0.44. rs11385942 was not evaluated in Minho Cohort. (PDF) [file pone.0264009.s001.pdf]
