# Supplementary material for: Evaluation of the genetic risk for COVID-19 outcomes in COPD and differences among worldwide populations
Source: PLoS One. 2022 Feb 23;17(2):e0264009. doi: 10.1371/journal.pone.0264009 (PMC8865687; doi:10.1371/journal.pone.0264009)
Supplement: S1 Table — Ref. allele—reference allele. Alt. allele—alternative allele. A—adenine. C—cytosine. G—guanine. T—thymine. dupA—duplication of an adenine. V—valine. M—Methionine. (PDF) [file pone.0264009.s002.pdf]

S1 Table. COVID-19 associated SNPs description. Ref. allele - reference allele. Alt. allele - alternative allele. A - adenine. C - cytosine. G - guanine. T - thymine. dupA - duplication of an adenine. V - valine. M - Methionine.

SNP	Position	Ref. allele	Alt. allele	Aminoacid alteration	Gene : Consequence	Clinical Significance
rs286914	chr11:34653124	G	A	No alteration	EHF : Intron Variant	Not Reported in ClinVar
rs12329760	chr21:41480570	C	T	V [GTG] > M [ATG]	TMPRSS2 : Missense Variant	Not Reported in ClinVar
rs657152	chr9:136139265	A	C,T	No alteration	ABO : Intron Variant	Not Reported in ClinVar
rs11385942	chr3:45876461	A	dupA	No alteration	LZTFL1 : Intron Variant	Not Reported in ClinVar
