# Supplementary material for: Evaluation of the genetic risk for COVID-19 outcomes in COPD and differences among worldwide populations
Source: PLoS One. 2022 Feb 23;17(2):e0264009. doi: 10.1371/journal.pone.0264009 (PMC8865687; doi:10.1371/journal.pone.0264009)
Supplement: S2 Table — SNP X and SNP Y are representative. p2—major allele in homozygosity probability; 2pq—heterozygosity probability; q2—minor allele in homozygosity probability. (PDF) [file pone.0264009.s003.pdf]

S2 Table. Probability formula of having multiple risk alleles, assuming the Hardy-Weinberg's law.

SNP X and SNP Y are representative. p2 - major allele in homozygosity probability; 2pq - heterozygosity probability; q2 - minor allele in homozygosity probability.

		SNP Y		
		p^2	2pq	q^2
SNP X	p^2	prob. 0 alleles	prob. 1 allele	prob. 2 alleles
	2pq	prob. 1 allele	prob. 2 alleles	prob. 3 alleles
	q^2	prob. 2 alleles	prob. 3 alleles	prob. 4 alleles
