# Supplementary material for: Evaluation of the genetic risk for COVID-19 outcomes in COPD and differences among worldwide populations
Source: PLoS One. 2022 Feb 23;17(2):e0264009. doi: 10.1371/journal.pone.0264009 (PMC8865687; doi:10.1371/journal.pone.0264009)
Supplement: S3 Table — N (%)—number of individuals and corresponding percentage; remaining data is presented as medians with interquartile range in square brackets. FEV1—Forced Expiratory Volume in 1-sec in litres; FVC—Forced Vital Capacity in litres; n.d.—no data available. (PDF) [file pone.0264009.s004.pdf]

S3 Table. Sociodemographic, anthropometric and clinical characteristics of the Minho cohort. N (%) - number of individuals and corresponding percentage; remaining data is presented as medians with interquartile range in square brackets. FEV1 - Forced Expiratory Volume in 1-sec in litres; FVC - Forced Vital Capacity in litres; n.d. - no data available.

**Characteristics**	**Minho**
	**Healthy (n=380)**
Age (years)	66 [53, 72]
Gender (Male), n (%)	183 (48.2%)
Body mass Index (Kg/m^2)	27.65 [25.1, 30.45]
FEV_1 (Litres)	n.d.
FEV_1/FVC	n.d.
