# Supplementary material for: Evaluation of the genetic risk for COVID-19 outcomes in COPD and differences among worldwide populations
Source: PLoS One. 2022 Feb 23;17(2):e0264009. doi: 10.1371/journal.pone.0264009 (PMC8865687; doi:10.1371/journal.pone.0264009)
Supplement: S4 Table — SNP—single nucleotide polymorphism; CHR:POS—genomic coordinates; N -total number of individuals enrolled in the study; A1—effect allele; A2—reference allele; A1F - Average effect allele frequency; A2F - Average reference allele frequency. Data source: rs286914, rs12329760 and rs11385942—gnomAD-Genome project7; rs657152—ALFA project6. (PDF) [file pone.0264009.s005.pdf]

S4 Table. Single-loci frequency comparison between European and the other world populations. SNP - single nucleotide polymorphism; CHR:POS - genomic coordinates; N -total number of individuals enrolled in the study; A1 - effect allele; A2 - reference allele; A1F - Average effect allele frequency; A2F - Average reference allele frequency. Data source: rs286914, rs12329760 and rs11385942 - gnomAD-Genome project7; rs657152 - ALFA project6.

Study Population	SNP	CHR:POS	N	A1	A2	A1F	A2F	p-value
European	rs286914	chr11:34653124	18876	A	G	26.81	73.19	---------
Portuguese			623	A	G	31.95	68.05	<0.0001
African			8682	A	G	38.01	61.99	<0.0001
American			846	A	G	35.9	64.1	<0.0001
East Asian			1560	A	G	13.14	86.86	<0.0001
European	rs12329760	chr21:42852497	18848	T	C	27.23	72.77	---------
Portuguese			623	T	C	18.74	81.26	<0.0001
African			8694	T	C	28.84	71.16	<0.0001
American			848	T	C	17.2	82.8	<0.0001
East Asian			1558	T	C	41.72	58.28	<0.0001
European	rs657152	chr9:136139265	82068	A	C	37.09	62.91	---------
Portuguese			623	A	C	42.14	57.86	<0.0001
Spanish			9761	A	C	37.41	62.59	0.383
Italian			6363	A	C	38.43	61.57	0.0025
African			2886	A	C	45.08	54.92	<0.0001
American			4098	A	C	22.65	77.35	<0.0001
East Asian			18	A	C	72	28	<0.0001
European	rs11385942	chr3:45876459	18896	GA	G	7.87	92.13	---------
Portuguese			243	GA	G	5.82	94.18	0.0594
Spanish			9761	GA	G	6	94	<0.0001
Italian			6363	GA	G	10.33	89.67	<0.0001
African			8704	GA	G	5.76	94.24	<0.0001
American			846	GA	G	5.8	94.2	0.0018
East Asian			1560	GA	G	0.06	99.94	<0.0001
