# Supplementary material for: Evaluation of the genetic risk for COVID-19 outcomes in COPD and differences among worldwide populations
Source: PLoS One. 2022 Feb 23;17(2):e0264009. doi: 10.1371/journal.pone.0264009 (PMC8865687; doi:10.1371/journal.pone.0264009)
Supplement: S5 Table — 0 to 4 represent the sum of effect alleles. Data for the Portuguese population correspond to observed values (n = 623) extrapolated to 1 million, whereas data for major world populations correspond to estimations (also to 1 million) based on the published effect allele frequencies, after Hardy-Weinberg equilibrium validation. Allele frequencies were obtained from gnomAD-Genome project7. (PDF) [file pone.0264009.s006.pdf]

S5 Table. Estimation on the number of people with a cumulative number of risk alleles in the world major populations, for susceptibility to COVID-19 infection (rs286914 + rs12329760). 0 to 4 represent the sum of effect alleles. Data for the Portuguese population correspond to observed values (n=623) extrapolated to 1 million, whereas data for major world populations correspond to estimations (also to 1 million) based on the published effect allele frequencies, after Hardy-Weinberg equilibrium validation. Allele frequencies were obtained from gnomAD-Genome project7.

		Risk				
		0	1	2	3	4
Population	European	283667	420110	233310	57584	5330
	Portuguese	295804	426664	223339	50112	4081
	Spanish	---------	---------	---------	---------	---------
	Italian	---------	---------	---------	---------	---------
	African	194588	396354	298545	98496	12017
	American	281693	432564	231605	50325	3813
	Asian	256259	444421	248188	48128	3005
