# Supplementary material for: Evaluation of the genetic risk for COVID-19 outcomes in COPD and differences among worldwide populations
Source: PLoS One. 2022 Feb 23;17(2):e0264009. doi: 10.1371/journal.pone.0264009 (PMC8865687; doi:10.1371/journal.pone.0264009)
Supplement: S6 Table — 0 to 4 represent the sum of effect alleles. Data for the Portuguese(n = 623), Spanish (n = 9761) and Italian (6363) populations correspond to observed values extrapolated to 1 million, whereas data for major world populations correspond to estimations (also to 1 million) based on the published effect allele frequencies, after Hardy-Weinberg equilibrium validation. Allele frequencies for rs11385942 were obtained from gnomAD-Genome project7, while rs657152 allele frequencies were obtained from the ALFA project6. (PDF) [file pone.0264009.s007.pdf]

S6 Table. Estimation on the number of people with a cumulative number of risk alleles in the world major populations for severe COVID-19 with respiratory failure (rs657152 + rs11385942). 0 to 4 represent the sum of effect alleles. Data for the Portuguese(n=623), Spanish (n=9761) and Italian (6363) populations correspond to observed values extrapolated to 1 million, whereas data for major world populations correspond to estimations (also to 1 million) based on the published effect allele frequencies, after Hardy-Weinberg equilibrium validation. Allele frequencies for rs11385942 were obtained from gnomAD-Genome project7, while rs657152 allele frequencies were obtained from the ALFA project6.

		Risk				
		0	1	2	3	4
Population	European	335924	453495	186890	22839	852
	Portuguese	292834	470920	215022	20660	564
	Spanish	346151	457978	177895	17472	504
	Italian	304812	450737	210465	32410	1576
	African	267875	472505	235241	23705	674
	American	530912	376306	85825	6785	173
	Asian	78306	402810	518262	622	0
