# Supplementary material for: Evaluation of the genetic risk for COVID-19 outcomes in COPD and differences among worldwide populations
Source: PLoS One. 2022 Feb 23;17(2):e0264009. doi: 10.1371/journal.pone.0264009 (PMC8865687; doi:10.1371/journal.pone.0264009)
Supplement: Attachment — Submitted filename: Response to Reviewers.docx [file pone.0264009.s009.docx]

**RESPONSE TO REVIEWERS**

**Manuscript Title:** “Genetic risk for COVID-19 outcomes in COPD and differences among worldwide populations”

**Manuscript ID:** PONE-D-21-21399

We are thankful to the editor and to the reviewers for the time they spent considering our article and for all their valuable comments. We believe that after addressing all comments and suggestions, the manuscript has improved. Please find below our point-by-point responses.

**Reviewer #1**

**--------------------------------------------------------------------------------------------------------------------------------------**

**Comments to Authors**

**Comment 1:** Fig. S3 should not come before Fig. S1 and Fig. S2.

**Response:** Figures S1 and 2 have now been integrated in the manuscript following reviewer’s suggestion below. Please see Figure 6 in the main manuscript.

**Comment 2:** The heatmap data of Fig. S1 and Fig. S2 can be considered as main figures.

**Response:** Following the reviewer´s suggestion, both figures are now main figures. Please see Figure 6 in the main manuscript.

**Comment 3:** All figures can be improved by providing extra specific information. For example, in Fig. 1, title like “SNPs for COVID-19 susceptibility” or “SNPs for severe COVID-19 response” can be added; In Fig. 2, indicate what the “0, 1, 2, 3, 4” represent; In Fig. 4 and Fig. 5, a label such as “data not available” should be included on the Spanish and Italian groups instead of leaving them blank (as this may represent 0).

**Response:** Thank you for this suggestion. Additional information was added as recommended and we believe figures are now more intuitive. Please see Figures 1 (page 11), 2 (page 12), 4 (page 16) and 5 (page 18).

**Comment 4:** Why write the p-values in the figure legends but not label them directly in the graphs?

**Response:** The statistical significance labels were not included initially in the graphs as we thought they would become too busy and difficult to interpret. After including the label on the graphs, we conclude that it does not make the graphs busier and we have now followed the reviewer’s suggestion. Please see Figures 4 and 5 (page 16 and 18 respectively).

**Comment 5:** In Figure 5, how are the p-values being calculated (which data compared with which)?

**Response:** P-values of figure 5 were calculated by means of a Chi-Square goodness of fit test, comparing each population’s risk distribution with the risk distribution of the European population (as stated in the methodology section). Please see page 9, line 164-167.

“*Chi-Square test was also used to assess differences in proportions between populations (European vs every other population) for the cumulative risk of having two SNPs, for either COVID-19 infection susceptibility or severe COVID-19 with respiratory failure*.”

**Comment 6:** Make sure the references are in the correct format (e.g. ref. 10 and 11).

**Response:** Please find references now corrected (page 24, line 401-405).

**Comment 7:** rs41303171 was mentioned in the introduction as a genetic variant possibly playing a role in COVID-19 susceptibility. However, why is this SNP not tested? Especially when the gene of this SNP encodes the most important SARS-CoV-2 receptor protein, ACE2 (DOI: 10.1002/RMV.2122).

**Response:** We fully agree with the reviewer, a SNP located in the ACE2 encoding gene is of most importance, given ACE2 known role in covid-19 infection pathway. Unfortunately, rs41303171 was neither in our genotyping arrays nor were we able to include it through imputation. Acknowledging the relevance of your comment we have now added this information to the study limitations section (page 22, line 355-357).

“*Rs41303171 may play a very important role in COVID-19 infection, knowing that this SNP is located in the gene encoding the most important SARS-CoV-2 receptor protein (ACE2) (37), however it could not be found in either of our cohorts, inhibiting its analysis*.”

**Comment 8:** It would be very helpful for the authors to include a table with SNP information (e.g. associated gene? Intron or exon variant? Missense or synonymous variant? Potential role in COVID-19 risk? etc.).

**Response:** Please find a table with the requested SNP information added to the supplementary material (S1 Table).

**Comment 9:** The authors claimed that the “people with COPD do not have increased genetic risk for COVID-19”, however, it has to be made clear that this statement is based on the assessments of only the selected 4 SNPs and limited study samples.

**Response:** Please find our statement made clearer in the conclusions (page 23, line 370-371).

*“**This study showed that genetic background does not play a significant role in predisposition to severe COVID-19 among people with COPD based on the explored genetic variants.”*

**Comment 10:** In addition to only concluding that “genetic backgrounds do not play a significant role in COVID-19 risk among people with COPD”, results obtained in Figures 4 and 5 are also very interesting, and should be further discussed/concluded. Indeed, several studies have shown that certain populations or ethnicities appear to have higher risk/severity of COVID-19 outcomes (e.g. DOI: 10.1017/cjn.2021.148).

**Response:** We have accepted the reviewer suggestion and further discussed the populational results in light of other studies. Please see page 22 lines 348 to 350.

**Reviewer #2**

**--------------------------------------------------------------------------------------------------------------------------------------**

**Comments to Authors**

**Comment 1:** Given that there is little genetic contribution for COVID-19 infection predisposition or worse outcomes observed in people with COPD, why are the authors not checking on populations with other lung-related diseases, such as asthma, ARDS, as well as tuberculosis?

**Response:** We do agree with the value of applying the same rationale of this work to other lung-related diseases. Unfortunately, we do not have access to samples from people with non-COPD condition.

**Comment 2:** The title might be overstated and should be more specific on the ethnicity of the population being studied.

**Response:** We thank the reviewer for the suggestion. The title has been altered to allow a more neutral read. Nevertheless, we would like to emphasise this is highly challenging as we report on two independent studies. The first study reports the differences in genetic risk for covid-19 associated outcomes between people with COPD and healthy people, where we feel that specifying the population as being Portuguese would not add relevant value, given that the focus is on having or not having COPD; The second study reports the genetic risk for covid-19 associated outcomes in different world populations, all of which are compared to the European (used as reference).

**Comment 3:** The current title looks like as if there is genetic risk of COPD patients to COVID-19, this should be amended.

**Response:** We agree with the reviewer that the title was misleading. We altered the title accordingly. Please see the new title.

*“Evaluation of the genetic risk for COVID-19 outcomes in COPD and differences among worldwide populations”*

**Comment 4:** For the SNPs already associated with susceptibility to COVID-19 infection/severe COVID-19 with respiratory failure, what exactly are these changes? E.g., any change of amino acids on encoded protein genes? These should be elaborated.

**Response:** A table (supplementary table 1) with the mentioned/requested information about the SNPs has been added, as requested by another reviewer too.

**Comment 5:** It is unclear on the applications of the current work.

**Response:** The major application of this work is unravelling the low genetic impact on the increased risk for covid-19 outcomes in people with COPD. Our work also comprises the methodology necessary to perform a comprehensive analysis of genetical factors (single-loci, bi-allelic and polygenic risk), which could be applied in other cohorts relevant to covid-19 or even other non-covid-19 related scenarios. Finally, we highlight the need for a “higher resolution map” specifically of the European populations, as all European sub-populations addressed in this study showed different genetic backgrounds.

**Comment 6:** Some of the figure details and text are too small to be seen.

**Response:** We have revised the figures and enlarged the details according to the reviewer’s suggestion. Please see Figures 4 and 5 (page 16 and 18 respectively).

**Comment 7:** Typos and unfriendly mode of English usage can be found.

**Response:** The article has been revised for typos and unfriendly mode of English usage.
